# Comparative machine learning analysis identifies random forest and adaboost as superior models for the evaluation of semen quality and reproductive hormones

**DOI:** 10.3389/frph.2026.1908391

**Published:** 2026-08-06

**Authors:** Shubhadeep Roychoudhury, Saurav Paul, Birupakshya Paul Choudhury, Sandipan Das, Kushal Kumar Kar, Ishani Bhattacharjee, Arun Paul Choudhury, Israel Maldonado Rosas, Saptarshi Paul

**Affiliations:** 1 Department of Life Science and Bioinformatics, Assam University, Silchar, India; 2 Department of Computer Science, Assam University, Silchar, India; 3 Mediland Hospital and Research Centre, Silchar, India; 4 Department of Biotechnology, Gurucharan University, Silchar, India; 5 Department of Obstetrics and Gynaecology, Silchar Medical College, Silchar, India; 6 Citmer Reproductive Medicine, Mexico City, Mexico

**Keywords:** diagnostic models, ensemble learning, kernel learning, linear learning, male hormonal panel, male infertility, sperm parameters

## Abstract

**Background:**

Semen analysis is a widely accepted laboratory investigation for evaluating male infertility. However, in cases of idiopathic or unexplained male infertility, comprehensive assessment may require blood-based profiling of a reproductive hormone panel—including follicle-stimulating hormone (FSH), luteinizing hormone (LH), prolactin (PRL), and testosterone—to clearly identify endocrine abnormalities that may contribute to infertility. These variables exhibit nonlinear dynamics and multidimensional interdependencies, presenting analytical challenges for conventional statistical approaches to detect subtle, latent patterns within such complex data. In contrast, advanced analytics such as machine learning (ML) algorithms enable robust modelling and interpretation of high-dimensional datasets.

**Methodology:**

Pre-processing of data included normalization and correlation analysis. Using a train-test split ratio of 80:20, nine ML algorithms—Linear Regression, Lasso Regression, Ridge Regression, Elastic Net, Random Forest, Support Vector Regression, Gradient Boosting, AdaBoost, and Neural Networks—were trained using cross-validation and hyperparameter optimization. Model performance was assessed using the coefficient of determination (R^2^), root mean square error (RMSE), and feature importance analysis.

**Results:**

Ensemble learning approaches consistently outperformed conventional regression models. AdaBoost achieved the highest predictive accuracy for sperm motility (R^2^ = 0.993), while Gradient Boosting yielded superior predictions for progressive motility (R^2^ = 0.932) and vitality (R^2^ = 0.982). Random Forest demonstrated the strongest performance for semen volume (R^2^ = 0.428), LH (R^2^ = 0.432), and PRL (R^2^ = 0.586). Conversely, predictions for seminal pH (R^2^ = 0.037), liquefaction time (R^2^ = 0.158), FSH (R^2^ = 0.178), and testosterone (R^2^ = −0.114) were limited. Feature importance analysis identified total sperm count, sperm concentration, non-progressive motility, morphology, and non-motile sperm percentage as the most influential predictors across models.

**Conclusions:**

Random Forest and AdaBoost emerged as the most effective and broadly applicable models for evaluating male reproductive parameters, whereas Gradient Boosting exhibited exceptional predictive capacity for select semen parameters.

## Introduction

Male infertility is defined as a man's inability to contribute to a natural pregnancy with a fertile female partner (1). Despite extensive research, male infertility remains a complex, multifactorial condition with significant heterogeneity, which makes diagnosis and prediction challenging (2, 3). It is estimated that about 8%–10% of couples are affected by infertility globally, and male factor infertility alone contributes to approximately 50% of all cases (2). Over recent decades, increasing evidence has shown a decline in semen quality, in particular sperm counts worldwide, further intensifying concerns regarding male reproductive health (1, 4). Declines in semen quality parameters, including semen volume, total sperm count, sperm concentration, motility, progressive motility, morphology, and vitality, have also been linked to lifestyle factors such as obesity, psychological stress, smoking, alcohol consumption, sedentary behaviour, and exposure to environmental toxins apart from underlying biological dysfunctions (5). Population-based studies indicate that approximately one-third of infertility cases are primarily attributed to male-related factors, while the remaining cases arise due to combined male and female contributions or remain classified as idiopathic and unexplained infertility (2, 6). Conventionally, semen analysis remains the first line laboratory investigation for assessing male fertility potential (7). Alterations in seminal characteristics are frequently associated with impaired reproductive performance and may serve as important indicators of hidden pathophysiological conditions (8). Sperm production and sexual development are also significantly influenced by hormones generated by the testicles, hypothalamus, and pituitary gland. In particular, the cases of idiopathic or unexplained infertility may involve such hidden abnormalities as altered hypothalamic-pituitary-gonadal (HPG) axis activity, metabolic imbalance, oxidative stress, or mild functional irregularities necessitating blood-based reproductive hormonal profiling (9, 10). Assessment of hormonal panel, including follicle-stimulating hormone (FSH), luteinizing hormone (LH), prolactin (PRL), and testosterone, may provide an important mechanistic overview into reproductive dysfunction and help identify endocrine abnormalities that may contribute to infertility even in the absence of obvious seminal alterations (5, 11). The identification of male infertility, therefore, requires a systematic diagnostic approach that evaluates both seminal and endocrine determinants of reproductive competence. Although laboratory-based semen analysis and hormonal evaluation provide valuable diagnostic information, they do not always yield definitive clinical interpretation, sometimes reducing diagnostic precision and complicating interpretation in idiopathic or unexplained infertility cases. To address the limitations of conventional diagnostic approaches, modern computational techniques such as machine learning (ML) have become powerful tools for analyzing intricate biological datasets (12). ML-based predictive models enable integrating seminal and hormonal variables to identify hidden patterns and capture non-linear relationships without relying on predefined statistical assumptions. These approaches are particularly useful in male infertility studies, where multiple interacting biological factors collectively influence fertility outcomes (13). Accordingly, several algorithms, including Linear Regression, Ridge Regression, Lasso Regression, Elastic Net, Random Forest, Gradient Boosting, AdaBoost, Support Vector Regression (SVR), and Neural Networks, have been widely applied for predictive modelling in male infertility. The present study attempted: (i) to assess the correlations between semen quality parameters and reproductive hormone levels using correlation mapping; and (ii) to develop and compare ML algorithms for predicting semen quality parameters and reproductive hormones. Therefore, the present study assessed the effectiveness of multiple ML algorithms in predicting semen quality parameters and key reproductive hormone levels in the study population.

## Materials and methods

### Study design and dataset

This investigation employed a retrospective study design to construct predictive models utilizing ML algorithms. Prior to analysis, the dataset—comprising seminal and hormonal parameters from male patients presenting with infertility issues at two tertiary healthcare centres in Silchar, northeast India—was systematically examined for missing values, outliers, and data inconsistencies. The final study population consisted of 104 participants (*n* = 104). Continuous variables were standardized using z-scores to ensure comparability across measures before model development. Ethical approval for the study was obtained from the Institutional Ethics Committee of Assam University, Silchar (IEC/AUS/2021/35/BPC, dated 26th February 2021). Informed oral and written consent was secured from all participants prior to their inclusion in the study.

### Machine learning models

The dataset was randomly split into training and test sets at an 80:20 ratio. All ML models were trained on the training set, while the test set was saved for final performance assessment. To improve model generalization and minimize overfitting, hyperparameters were tuned using k-fold cross-validation (k = 5) on the training data. The best hyperparameter settings, as determined by cross-validation, were then used to train the final models. The independent test set, which was not involved in training or tuning, provided an unbiased evaluation of the models' predictive performance.

### Model evaluation and validation

The root mean square error (RMSE) and coefficient of determination (R^2^) were used to assess the model performance (14). Scatter plots depicting observed vs. predicted values were generated to evaluate model fit. Residual analyses were conducted to identify systematic patterns in prediction errors, with particular attention to heteroscedasticity. Cross-validation outcomes were reviewed to determine the consistency of model performance across validation folds (15).

### Normalization and preprocessing

Missing values in numeric predictors were imputed using the median, and categorical predictors were imputed using the mode. Label-encoded categorical variables were transformed using LabelEncoder on the entire pre-split dataset. After preprocessing, the dataset was split into 80% for training and 20% for hold-out testing (random_state = 42). Normalization was performed for models requiring scaled inputs, such as Linear Regression, Ridge, Lasso, Elastic Net, and SVR, using StandardScaler for z-score standardization. The scaler was fitted only on the training data before transforming the test set. Tree-based models (Random Forest, Gradient Boosting, AdaBoost) were trained on unscaled training features.

### Hyperparameter optimization and cross-validation

To ensure consistent comparison across all variables, each ML algorithm was implemented with a predetermined set of hyperparameters established before training. The same parameter configurations were used for all nine ML models throughout the study. The dataset was first split into training (80%) and independent test (20%) subsets using an 80:20 train–test split (test_size = 0.2). Model performance and stability were assessed via five-fold cross-validation (cv = 5) performed solely on the training data, with the R² serving as the evaluation metric. To ensure reproducibility, a fixed random seed (random state = 42) was set for both the train–test split and all stochastic algorithms that involve random initialization.

### Feature importance analysis

Predictor importance was evaluated using the mean decrease in impurity, determined by the impurity-based feature importance method from the Random Forest model. This metric represents the average reduction in impurity achieved by splits on each predictor across all trees in the ensemble, with higher values indicating a greater contribution to the model's decision-making process.

## Results

### Distribution of semen quality parameters and reproductive hormones

**Figure 1:**
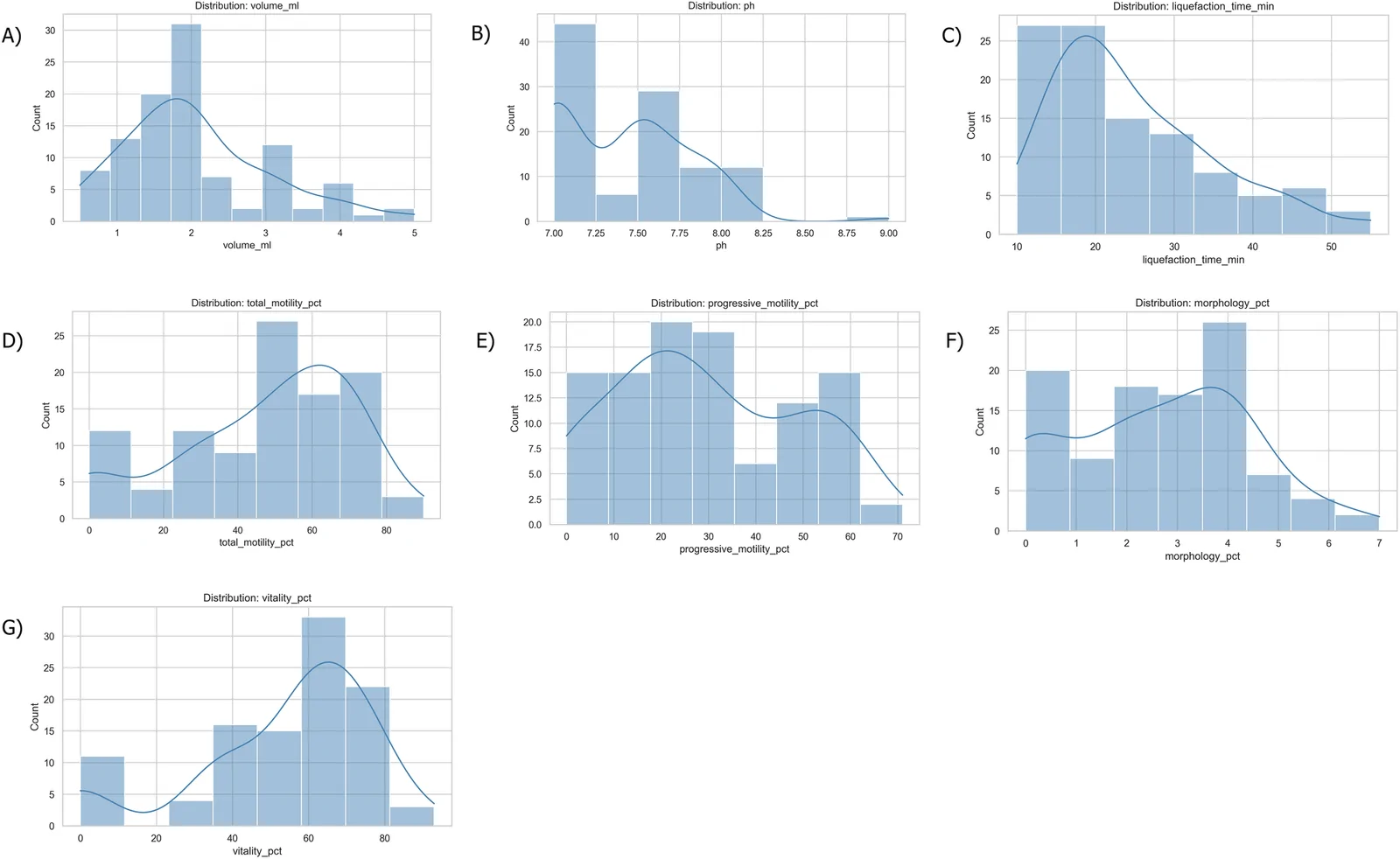
Distribution of semen quality parameters in the study population. **(A)** Semen volume exhibited a moderately right-skewed distribution, with most samples ranging from 1.0 to 2.0 mL; **(B)** Seminal pH showed low variability and remained largely within the physiological range, with a slight bimodal tendency; **(C)** Liquefaction time showed a positively skewed distribution, with most samples liquefying within 15–30 min; **(D)** Total sperm motility was predominantly distributed between 40% and 80%, indicating generally moderate-to-high motility among participants; **(E)** Progressive sperm motility displayed a broader distribution with evidence of bimodality; **(F)** Normal sperm morphology showed an approximately bell-shaped distribution with moderate positive skewness; and **(G)** sperm vitality exhibited a near-normal distribution with most observations clustered between 50% and 80%.

**Figure 2:**
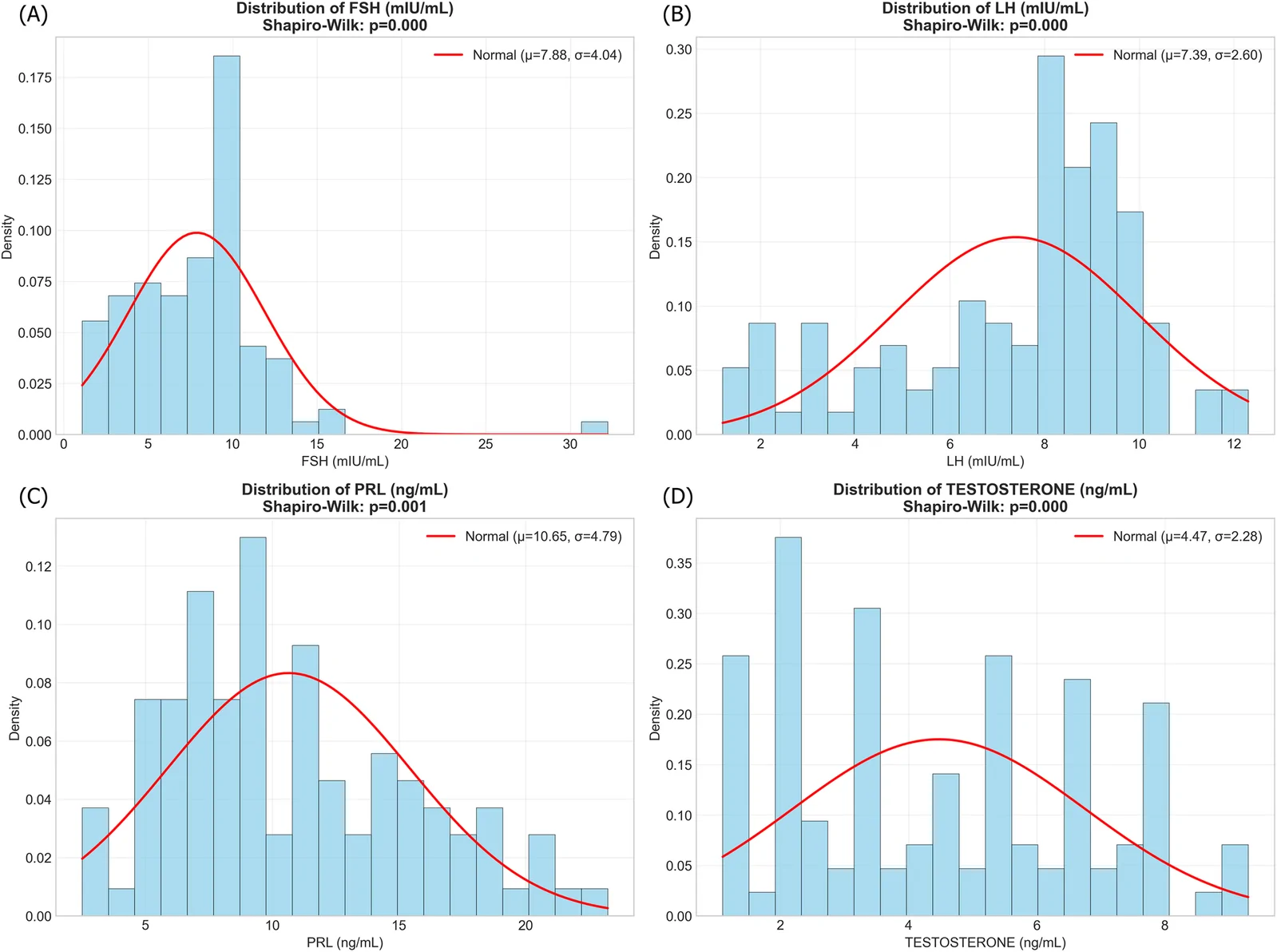
Distribution of reproductive hormones in the study population. **(A)** Follicle-stimulating hormone (FSH) exhibited a positively skewed distribution, with most samples concentrated between 4 and 12 mIU/mL and a few observations extending beyond 20 mIU/mL; **(B)** Luteinizing hormone (LH) was primarily distributed between 4 and 12 mIU/mL, with the highest density around 7–9 mIU/mL, although several lower and higher values contributed to a broader distribution; **(C)** Prolactin (PRL) demonstrated considerable variability, with most samples ranging from 5 to 15 ng/mL and a few elevated above 18 ng/mL, resulting in a moderately right-skewed distribution; and **(D)** Testosterone ranged from approximately 1–9 ng/mL, with most values distributed between 3 and 7 ng/mL. The highest frequency was observed around 4–5 ng/mL. Shapiro–Wilk tests indicated significant deviations from normality for all measured hormones (FSH: *p* < 0.001; LH: *p* < 0.001; PRL: *p* = 0.001; testosterone: *p* < 0.001).

Descriptive analysis of semen parameters and reproductive hormones revealed considerable variability among study participants. The mean semen volume was 2.06 ± 0.98 mL, demonstrating substantial inter-individual differences. The average liquefaction time was 24.97 ± 10.41 min, and the mean seminal pH was 7.41 ± 0.38. Sperm vitality averaged 54.00 ± 23.17%, with total sperm motility at 47.50 ± 23.63% and progressive motility with a mean value of 29.52 ± 19.43%. The mean sperm concentration was 40.55 ± 38.72 million/mL, and the average total sperm count was 90.75 ± 111.71 million per ejaculate. Mean sperm morphology was 2.62 ± 1.83%, indicating suboptimal morphology within this study population. Regarding reproductive hormones, the mean FSH level was 7.88 ± 4.03 mIU/mL, LH was 7.39 ± 2.59 mIU/mL, PRL was 10.64 ± 4.79 ng/mL, and testosterone was 4.47 ± 2.27 ng/mL (Supplementary Table S1). Semen volume has been found to be slightly right-skewed. Most samples ranged from 1.0 to 2.0 mL, with the highest frequency between 1.5 and 2.0 mL. However, a few samples reached volumes up to 5.0 mL. Seminal pH showed less variation than other semen parameters. Most samples were within the normal range, with values from 7.0 to 8.2. There were two small peaks around 7.1–7.2 and 7.5–7.7, suggesting a slight bimodal distribution. Liquefaction time showed that most semen samples liquefied within 15–30 min, while some took more than 45 min. Most of the samples exhibited moderate to high sperm motility, while a small number of samples displayed reduced motility. The distribution for total sperm motility was mostly concentrated between 40% and 80%, with a peak around 60%–70%. A small subset of samples showed slightly negative skewness, with low sperm motility values ranging from 0% to 20%. In comparison, the distribution of progressive motility ranged from 0% to 70%, with two minor peaks at 20%–30% and 50%–60%, indicating a bimodal pattern. The distribution of sperm morphology appeared bell-shaped, with moderate positive skewness. The distribution mainly ranged from 2% to 5%, with the highest frequency observed around 3%–4%. In the case of sperm vitality, a small number of samples had vitality below 20%. However, most of the samples were concentrated between 50% to 80%, with the highest density around 60% to 70%, demonstrating a near-normal distribution pattern (Figure 1). The distribution patterns of reproductive hormones showed considerable variability across samples. FSH concentrations exhibited a positively skewed distribution, with most samples ranging from 4 to 12 mIU/mL. The highest frequency was observed around 6–10 mIU/mL, while a small number of individuals displayed elevated FSH levels exceeding 20 mIU/mL. A significant deviation from normality was confirmed by the Shapiro–Wilk test (*p* < 0.001). LH concentrations ranged from 4 to 12 mIU/mL, with the highest density observed at 7–9 mIU/mL. Although most samples were clustered within this range, several lower and higher values contributed to the non-normal distribution. The Shapiro–Wilk test indicated a significant deviation from normality (*p* < 0.001). PRL levels showed considerable variability, with most samples ranging from 5 to 15 ng/mL. The highest frequency of PRL values was observed between 8 and 12 ng/mL, although a small number of samples showed concentrations above 18 ng/mL. The Shapiro–Wilk test indicated that the distribution of PRL values deviated significantly from normality (*p* = 0.001). Testosterone concentrations ranged from approximately 1 to 9 ng/mL, with most values falling between 3 and 7 ng/mL. The highest frequency was observed around 4–5 ng/mL. A small number of samples had relatively low or high testosterone concentrations, resulting in a wider distribution of testosterone concentrations. The Shapiro–Wilk test also indicated a significant deviation from normality (*p* < 0.001) (Figure 2).

### Correlation analysis

**Figure 3:**
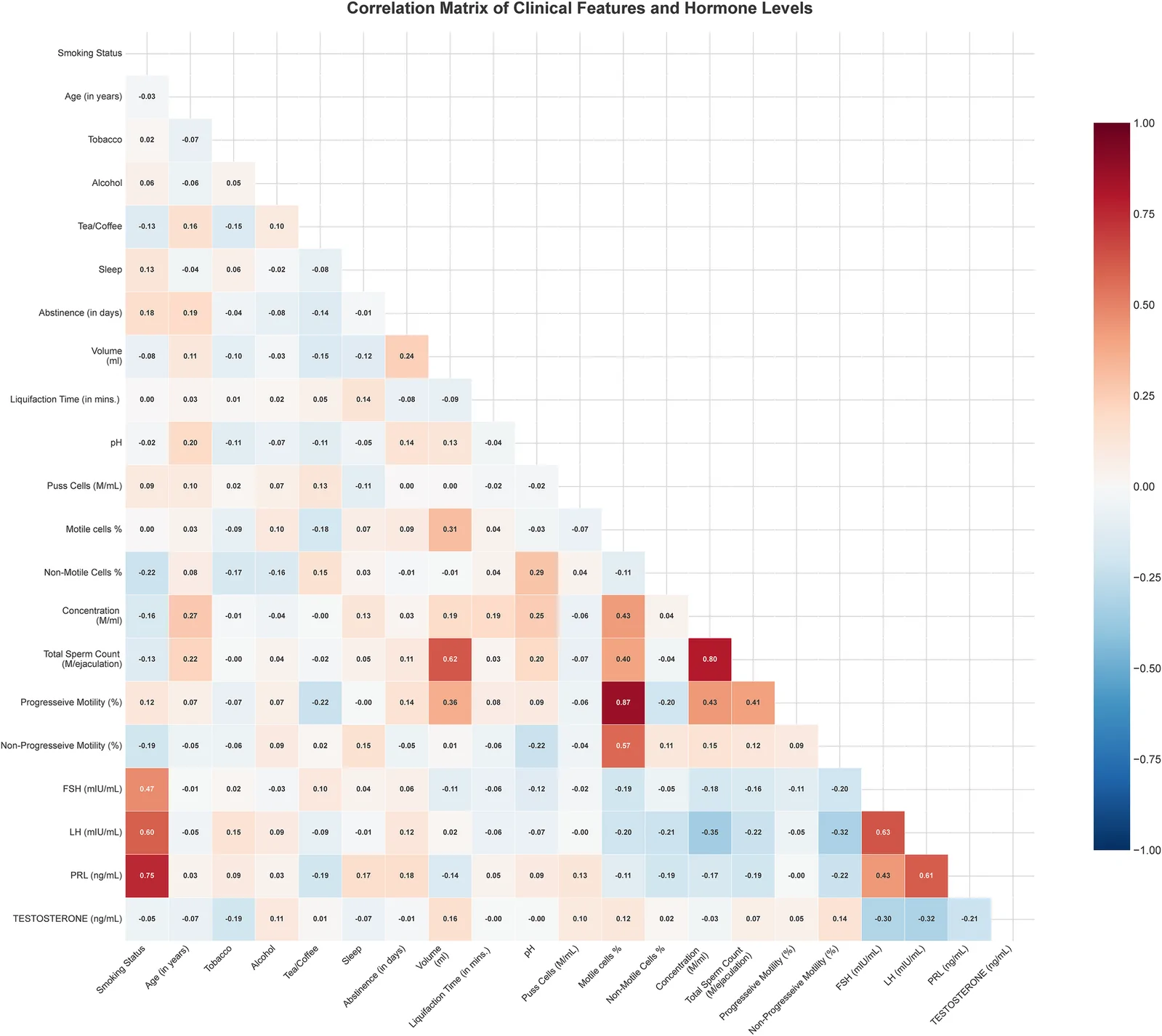
The correlation matrix shows the relationships among clinical variables, semen parameters, and reproductive hormone levels. The heatmap provides pairwise correlation coefficients for lifestyle factors such as smoking status, age, tobacco use, alcohol consumption, tea/coffee intake, sleep duration, and abstinence period. It also includes semen parameters like volume, liquefaction time, pH, pus cells, sperm motility, non-motile cells, sperm concentration, progressive motility, total sperm count, and non-progressive motility. Additionally, it features hormones including luteinizing hormone (LH), prolactin (PRL), testosterone, and follicle-stimulating hormone (FSH). Positive correlations are represented in hues of red, while negative correlations are depicted in shades of blue. The intensity of the colour reflects the strength of the relationship. Significant positive correlations were noted between progressive motility and total sperm motility (r = 0.87), total sperm count and sperm concentration (r = 0.80), semen volume and total sperm count (r = 0.62), FSH and LH levels (r = 0.63), as well as between LH and PRL (r = 0.61). Moderate positive correlations were identified between FSH and PRL (r = 0.43), total sperm motility and sperm concentration (r = 0.43), progressive motility and sperm concentration (r = 0.43), progressive motility and total sperm count (r = 0.41), and total sperm motility and total sperm count (r = 0.40). Negative associations were observed between LH and sperm concentration (r = −0.35), testosterone and LH (r = −0.32), LH and non-progressive motility (r = −0.32), testosterone and FSH (r = −0.30), progressive motility and non-motile sperm cells (r = −0.22), and tea/coffee consumption and progressive motility (r = −0.22).

Correlation analysis revealed varying degrees of association among lifestyle factors, semen quality parameters, and reproductive hormones (Figure 3). Among semen quality indicators, the strongest positive correlations were observed between sperm progressive motility and total motility percentage (r = 0.87), total sperm count and sperm concentration (r = 0.80), and semen volume and total sperm count (r = 0.62). Moderate positive correlations were observed between total motility and sperm concentration (r = 0.43), progressive motility and sperm concentration (r = 0.43), progressive motility and total sperm count (r = 0.41), and total motility and total sperm count (r = 0.40). Semen volume exhibited positive correlations with progressive motility (r = 0.36), total motility (r 0.31), concentration (r = 0.19), and abstinence period (r = 0.24). Age demonstrated weak positive correlations with sperm concentration (r = 0.27), total sperm count (r = 0.22), seminal pH (r = 0.20), and abstinence period (r = 0.19). Tea/coffee consumption showed weak negative associations with progressive motility (r = −0.22), total motility (r = −0.18), semen volume (r = −0.15), and seminal pH (r = −0.11). Liquefaction time showed weak correlations with other variables, with the strongest positive association with sleep duration (r = 0.14). Among reproductive hormones, strong positive correlations were observed between FSH and LH (r = 0.63), and between LH and PRL (r = 0.61). FSH and PRL were moderately positively correlated (r = 0.43). Smoking status exhibited positive correlations with FSH (r = 0.47), LH (r = 0.60), and PRL (r = 0.75). In comparison, testosterone showed negative correlations with FSH (r = −0.30), LH (r = −0.32), and PRL (r = −0.21). Several hormonal parameters were negatively associated with semen quality indicators. FSH showed negative correlations with total motility (r = −0.19), concentration (r = −0.18), total sperm count (r = −0.16), and non-progressive motility (r = −0.20). Similarly, LH was negatively correlated with sperm concentration (r = −0.35), total sperm count (r = −0.22), total motility (r = −0.20), and non-progressive motility (r = −0.32). Overall, semen quality parameters showed stronger interrelationships than lifestyle factors, whereas reproductive hormones showed moderate associations with both semen characteristics and behavioural variables.

### Machine learning model performance

The ML models' predictive capabilities were assessed using various semen parameter metrics, including volume, pH, liquefaction duration, total sperm motility, progressive motility, and vitality. The effectiveness of the models was measured using R^2^, RMSE, mean absolute error (MAE), and mean cross-validation R^2^. For semen volume, Random Forest performed best (R^2^ = 0.428; RMSE = 0.603; MAE = 0.437). Elastic Net and Lasso Regression demonstrated reduced effectiveness, resulting in R^2^ values of 0.375 and 0.353, respectively. For seminal pH, all models performed poorly. The highest R^2^ was obtained with Elastic Net (R^2^ = 0.037), while the remaining models produced negative R^2^ values. For liquefaction time, AdaBoost achieved the highest R^2^ (0.158), followed closely by Random Forest (0.155), although RMSE remained high (>8 min). For total sperm motility, AdaBoost performed best (R^2^ = 0.993; RMSE = 2.046; MAE = 1.493). Random Forest and Gradient Boosting showed excellent performance, with R^2^ values exceeding 0.98 and cross-validation R^2^ values ranging from 0.869 to 0.887. For progressive motility, Gradient Boosting achieved the highest performance (R^2^ = 0.932; RMSE = 5.265; MAE = 3.343), followed by Random Forest (R^2^ = 0.889) and AdaBoost (R^2^ = 0.874). Morphology prediction showed moderate performance. Gradient Boosting performed best, with an R^2^ of 0.414, RMSE of 1.172, and MAE of 0.895. Random Forest and Lasso Regression also delivered satisfactory predictive results. Vitality prediction showed outstanding performance, particularly with ensemble learning models. Gradient Boosting achieved the highest R^2^ score of 0.982, accompanied by an RMSE of 3.217 and an MAE of 2.480. Random Forest and AdaBoost also showed strong predictive accuracy, with R^2^ scores above 0.93 (Table 1).

**Table 1:** Best-performing machine learning (ML) model for semen parameters (semen volume, pH, liquefaction time, sperm motility, progressive motility, morphology and vitality) based on the coefficient of determination (R^2^) and percentage of variance.

Semen parameter	Best model	R^2^ score	Variance explained
Volume	Random Forest	0.428	42.8%
pH	Elastic Net	0.037	3.7%
Liquefaction Time	AdaBoost	0.158	15.8%
Sperm Motility	AdaBoost	0.993	99.3%
Progressive Motility	Gradient Boosting	0.932	93.2%
Morphology	Gradient Boosting	0.414	41.4%
Vitality	Gradient Boosting	0.982	98.2%

The predictive performance of ML models varied considerably among the evaluated reproductive hormones (Table 2). Random Forest demonstrated moderate performance for LH, achieving an R^2^ of 0.432 and an explained variance of 43.2%. PRL demonstrated the highest predictability among all evaluated hormones. The Random Forest model achieved an R^2^ score of 0.586, explaining approximately 58.6% of the total variance in PRL concentrations. In comparison, FSH exhibited lower predictive performance. AdaBoost produced the optimal model, with an R^2^ score of 0.178 and an explained variance of only 17.8% for FSH. Testosterone showed the poorest predictive performance among the evaluated hormones. The best-performing model, Random Forest, yielded a negative R^2^ of −0.114.

**Table 2:** Top performing machine learning (ML) models for the prediction of reproductive hormones: luteinizing hormone (LH), prolactin (PRL), follicle-stimulating hormone (FSH) and testosterone.

Hormone	Best model	R^2^ score	Variance explained
LH	Random Forest	0.432	43.2%
PRL	Random Forest	0.586	58.6%
FSH	AdaBoost	0.178	17.8%
Testosterone	Random Forest	−0.114	Not predictable with current features

The table summarizes the optimal model for each hormone, based on the coefficient of determination (R^2^) and the proportion of variance.

### Comparison across models

**Figure 4:**
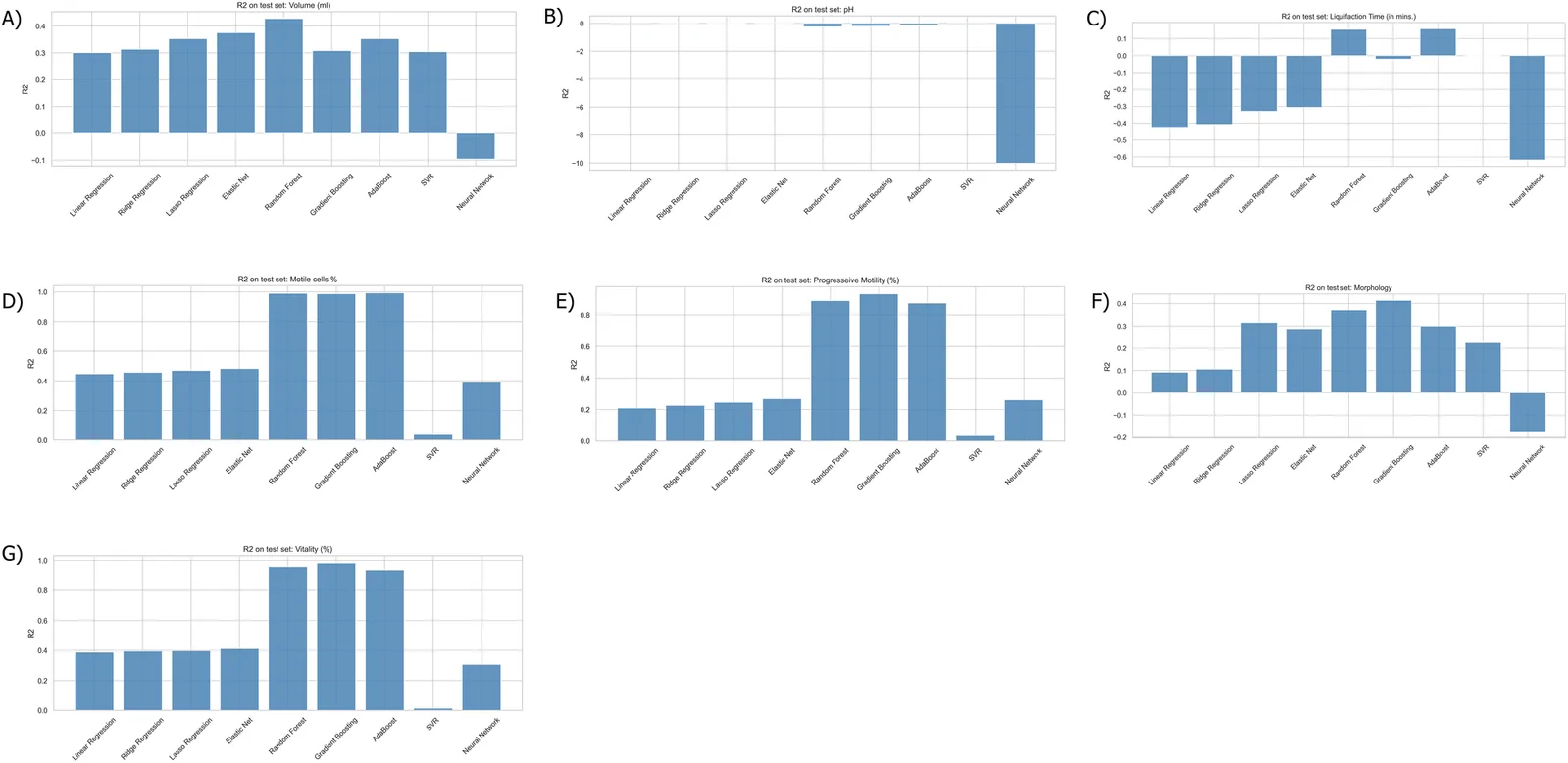
Comparison of R^2^ scores across machine learning (ML) algorithms for the prediction of semen parameters. The predictive abilities of Linear Regression, Ridge Regression, Lasso Regression, Elastic Net, Random Forest, Gradient Boosting, AdaBoost, Support Vector Regression (SVR), and Neural Network models were assessed. **(A)** The prediction of semen volume showed moderate performance across models, with R^2^ values ranging from 0.30 to 0.44. Random Forest achieved the highest R^2^ of 0.43; **(B)** The prediction of seminal pH demonstrated very low performance across all models, with R^2^ values close to zero. The Neural Network model even had a negative R^2^; **(C)** The prediction of liquefaction time showed poor overall performance. Most models produced negative R^2^ values. Random Forest and AdaBoost yielded small positive values but remained low; **(D)** The prediction of total sperm motility showed strong performance for ensemble methods. Random Forest, Gradient Boosting, and AdaBoost had R^2^ values close to 1.0. In contrast, linear models performed moderately, while SVR performed poorly; **(E)** The prediction of progressive motility followed a similar trend. Gradient Boosting (R^2^ = 0.95), Random Forest (R^2^ = 0.94), and AdaBoost (R^2^ = 0.94) performed better than other models; **(F)** The prediction of morphology showed moderate performance, with Gradient Boosting achieving the highest R^2^ value of 0.40. The Neural Network model showed negative performance; **(G)** The prediction of sperm vitality had the best overall performance. Gradient Boosting (R^2^ = 0.98), Random Forest (R^2^ = 0.95), and AdaBoost (R^2^ = 0.93) delivered the highest values.

**Figure 5:**
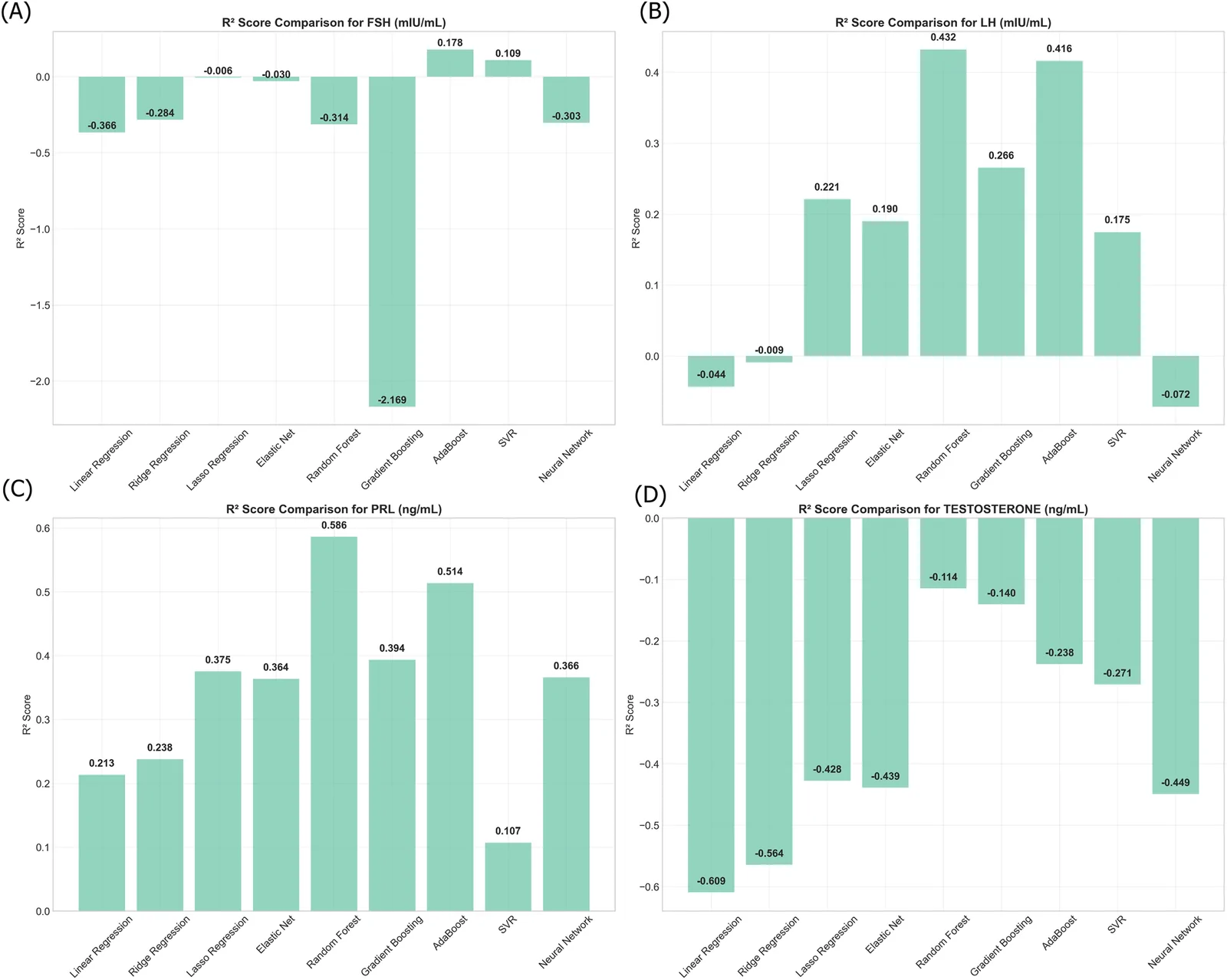
Comparison of coefficient of determination (R^2^) scores across nine machine learning algorithms for prediction of reproductive hormone levels. **(A)** FSH prediction showed poor performance across most models, with predominantly negative R^2^ values; AdaBoost and SVR showed the highest positive values, although performance remained low. **(B)** LH prediction showed improved performance, with Random Forest and AdaBoost achieving the highest R^2^ values, while linear regression-based models and the Neural Network performed worse. **(C)** PRL prediction showed the best overall performance, with all models producing positive R^2^ values and Random Forest and AdaBoost performing best. **(D)** Testosterone prediction showed poor performance across all models, with all algorithms producing negative R^2^ values.

Model performance varied considerably across semen parameters, with ensemble learning methods (Random Forest, Gradient Boosting, and AdaBoost) performing better than linear (Linear Regression, Lasso Regression, Ridge Regression, and Elastic Net) and kernel-based (SVR) models used in the present study. With R^2^ values ranging from 0.30 to 0.44, all models showed reasonable prediction power for semen volume. Random Forest performed the best among them, with an R^2^ of 0.43. For pH, all models demonstrated extremely low predictive capability, with R^2^ values close to zero. In particular, the Neural Network performed poorly, with a strong negative R^2^ value. Similarly, most models demonstrated negative R^2^ values for liquefaction time. Random Forest and AdaBoost showed positive R^2^ values; however, their prediction performance remains relatively low. In the case of total motility, Random Forest, Gradient Boosting and AdaBoost achieved R^2^ values close to 1.0. Linear models demonstrated moderate predictive accuracy, with an R^2^ of 0.45–0.48, while SVR exhibited low predictive performance, close to zero. Progressive motility demonstrated similar patterns, with Gradient boosting, Random Forest and AdaBoost producing R^2^ values of 0.95, 0.94 and 0.94, respectively. Linear regression models demonstrated moderate predictive performance, while SVR performed poorly. For sperm morphology, all models showed moderate predictive performance, with Gradient Boosting achieving the highest R^2^ of 0.40, while the Neural Network showed a negative R^2^. Sperm vitality prediction showed better overall model performance, with Gradient Boosting, Random Forest, and AdaBoost displaying R^2^ values of 0.98, 0.95, and 0.93, respectively. The Linear Regression model displayed moderate predictive performance, while the SVR showed negligible predictive power (Figure 4). Model performance, measured by R^2^ scores, varied across hormonal parameters. For hormones, too, ensemble methods generally performed better than linear and kernel-based models used in the present study. For FSH, all models had very low predictive performance. Most models produced negative R^2^ values, with Gradient Boosting performing the worst (R^2^ = −2.169). AdaBoost and SVR showed small positive R^2^ values of 0.178 and 0.109, respectively, but overall performance remained weak. For LH, Random Forest and AdaBoost achieved the best results, with R^2^ values of 0.432 and 0.416, respectively. Lasso Regression and Elastic Net showed moderate performance, while Linear Regression, Ridge Regression, and the Neural Network produced low or negative R^2^ values. For PRL, all models produced positive R^2^ values. Random Forest and AdaBoost performed best, with R^2^ values of 0.586 and 0.514, respectively, while linear models and the Neural Network showed moderate performance. SVR had the lowest performance, with an R^2^ of 0.107. For testosterone, all models performed poorly, with negative R^2^ values across methods. Random Forest and Gradient Boosting had the least negative values (R^2^ values of −0.114 and −0.140, respectively). Linear models performed worst, yielding R^2^ values ranging from −0.428 to −0.609 (Figure 5).

**Figure 6:**
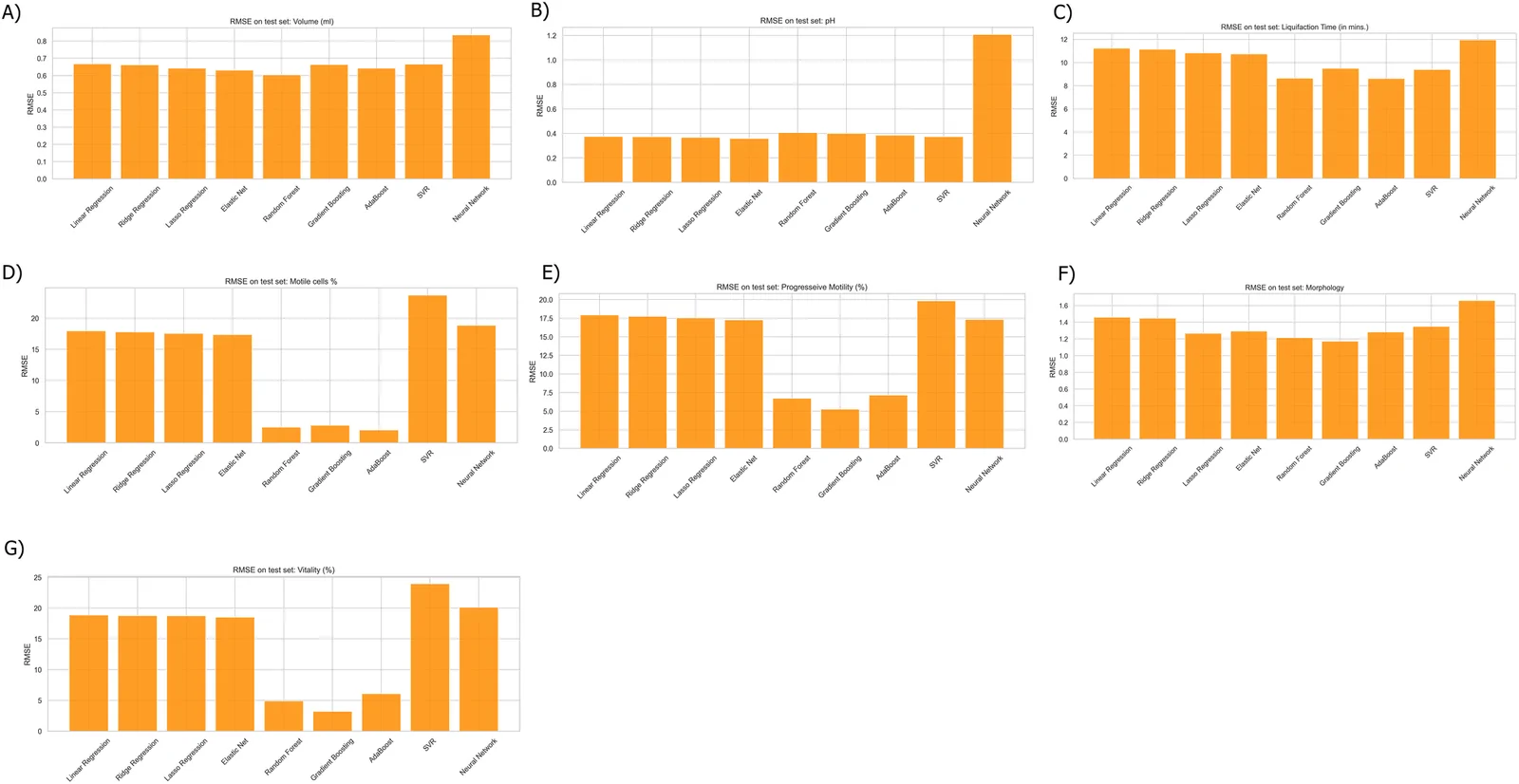
Comparison of root mean square error (RMSE) scores across machine learning (ML) algorithms for predicting semen parameters. The performance of Linear Regression, Ridge Regression, Lasso Regression, Elastic Net, Random Forest, Gradient Boosting, AdaBoost, Support Vector Regression (SVR), and Neural Network models was evaluated using. Lower values indicate better performance. **(A)** Semen volume prediction showed RMSE values from 0.60 to 0.84. Random Forest performed best with an RMSE of 0.60, while the Neural Network performed the worst; **(B)** Seminal pH prediction showed similar performance across most models, with RMSE around 0.38. The Neural Network had a higher error, exceeding 1.2; **(C)** Liquefaction time prediction showed a higher overall error, with RMSE around 11. Random Forest and AdaBoost performed best, with RMSE around 8.5; **(D)** Total sperm motility prediction had lower errors for ensemble methods. Random Forest, Gradient Boosting, and AdaBoost achieved RMSE values below 3.0. In contrast, linear models had higher errors, ranging from 17 to 18, and SVR performed the worst with an RMSE of 23; **(E)** Progressive motility showed a similar pattern. Gradient Boosting had an RMSE of about 5.0, Random Forest had an RMSE of 6.5, and AdaBoost had an RMSE of 7.0. These models outperformed linear regression and SVR; **(F)** Sperm morphology prediction showed moderate performance overall. Gradient Boosting had the lowest RMSE at 1.15, whereas the Neural Network had the highest error, exceeding 1.6; and **(G)** Vitality prediction had lower errors for ensemble methods. Gradient Boosting had an RMSE of about 3.0, Random Forest had an RMSE of 4.8, and AdaBoost had an RMSE of about 6.0. Linear models and SVR showed higher errors.

**Figure 7:**
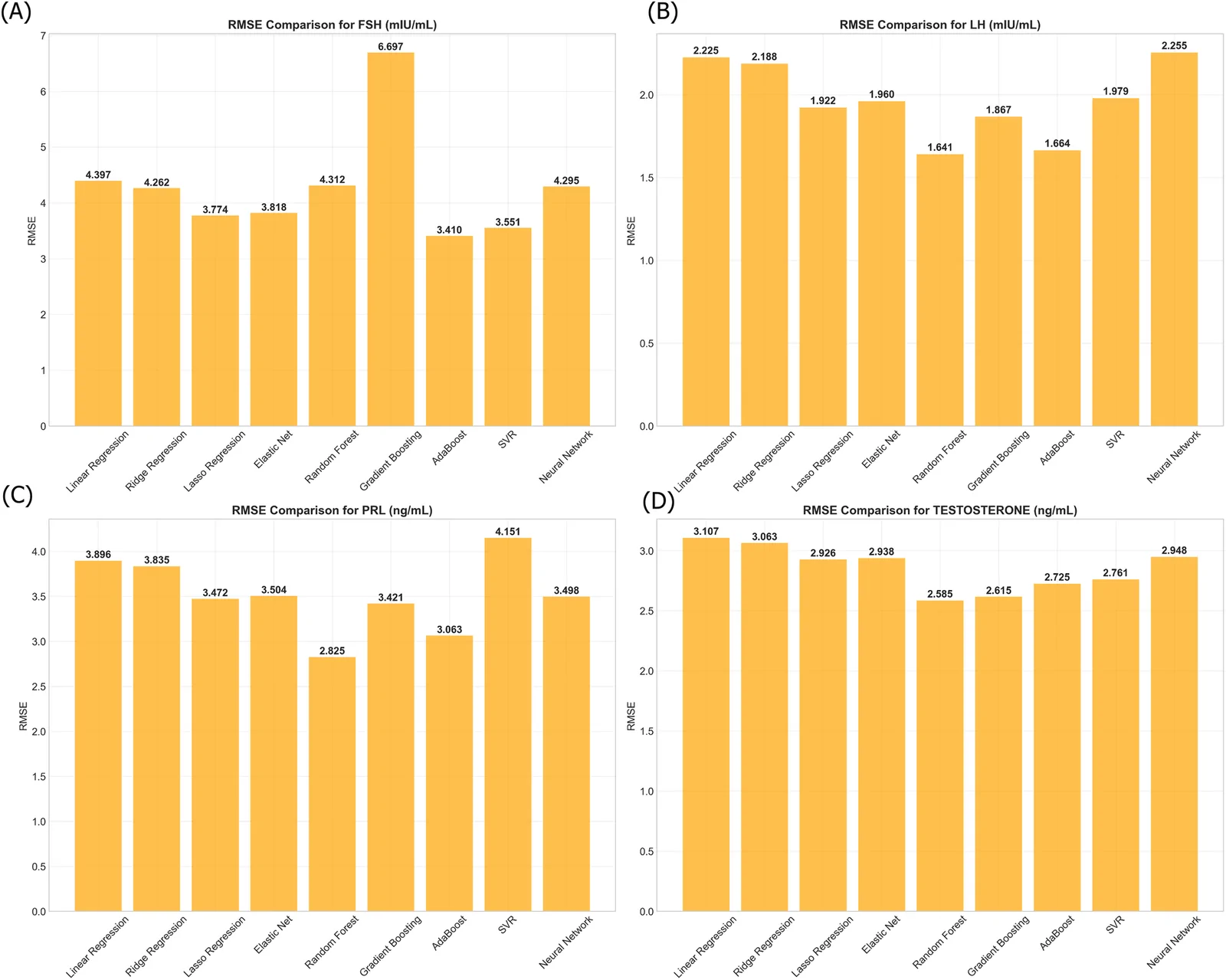
RMSE comparison for hormone prediction: comparison of root mean squared error (RMSE) across machine learning (ML) models for the prediction of reproductive hormones. Lower RMSE values indicate better performance. **(A)** Follicle-stimulating hormone (FSH) prediction showed mixed results across models. AdaBoost and Support Vector Regression (SVR) had the lowest errors, while Gradient Boosting had a higher RMSE than the others; **(B)** LH prediction performed better overall. Random Forest, Gradient Boosting, and AdaBoost achieved the lowest RMSE values, while linear regression models and the Neural Network had higher errors; **(C)** PRL prediction demonstrated strong performance for ensemble methods. Random Forest and AdaBoost achieved the lowest RMSEs, while SVR had the highest error; **(D)** Testosterone prediction showed lower errors for ensemble methods. Random Forest, Gradient Boosting, and AdaBoost produced the lowest RMSE values, while the Linear Regression model had the highest errors.

The assessment of model performance using RMSE revealed differences across semen parameters, with ensemble methods demonstrating lower errors than linear and kernel-based models. For semen volume, RMSE values ranged from 0.60 to 0.84 across models. Random Forest performed best (RMSE = 0.60), while the Neural Network performed worst. For pH, most models performed similarly, with RMSE values around 0.38; however, the Neural Network showed higher error (RMSE > 1.2). Liquefaction time showed higher error overall, with most models around RMSE ≈ 11. Random Forest and AdaBoost performed best (RMSE ≈ 8.5), although overall errors remained relatively high. For total sperm motility, Random Forest, Gradient Boosting, and AdaBoost achieved lower RMSE values (<3.0). Linear models showed higher prediction error, with RMSE values around 17–18, while SVR had the highest error (RMSE ≈ 23). Progressive motility showed a similar pattern, with Gradient Boosting, Random Forest, and AdaBoost producing lower RMSE values of approximately 5.0, 6.5, and 7.0, respectively. The Linear Regression model showed higher errors, around 17.5. SVR performed poorly with an RMSE of about ≈20. For sperm morphology, model performance was moderate overall. Gradient Boosting achieved the lowest RMSE of 1.15, while the Neural Network had the highest error (>1.6). For sperm vitality, Gradient Boosting, Random Forest, and AdaBoost produced lower RMSE values, approximately 3.0, 4.8, and 6.0, respectively. In contrast, linear models exhibited higher errors, around 18.5, and SVR had the highest RMSE, about 24. Overall, RMSE results showed that Gradient Boosting, Random Forest, and AdaBoost generally outperformed traditional regression models across most semen parameters, as illustrated in Figure 6. Model performance, assessed by RMSE, varied across hormonal parameters. Ensemble methods generally had lower errors than linear models, though there were some exceptions. For FSH, AdaBoost, and SVR, the lowest errors were achieved, with RMSE values of 3.410 and 3.551, respectively. Lasso Regression and Elastic Net performed moderately, while Gradient Boosting had the highest error, with an RMSE of 6.697. For LH, Gradient Boosting, Random Forest, and AdaBoost performed best, with RMSE values of 1.867, 1.641, and 1.664, respectively. Linear Regression and the Neural Network had higher errors, both above 2.2. For PRL, ensemble methods again showed lower errors. Random Forest and AdaBoost reached RMSE values of 2.825 and 3.063, respectively. Linear models and the Neural Network showed moderate errors, while SVR recorded the highest error with an RMSE of 4.151. For testosterone, Random Forest, Gradient Boosting, and AdaBoost had the lowest errors, with RMSE values of 2.585, 2.615, and 2.725, respectively. SVR also had a relatively low error at 2.761, while Linear Regression had the highest error with an RMSE of 3.107 (Figure 7).

### Actual vs. predicted values

**Figure 8:**
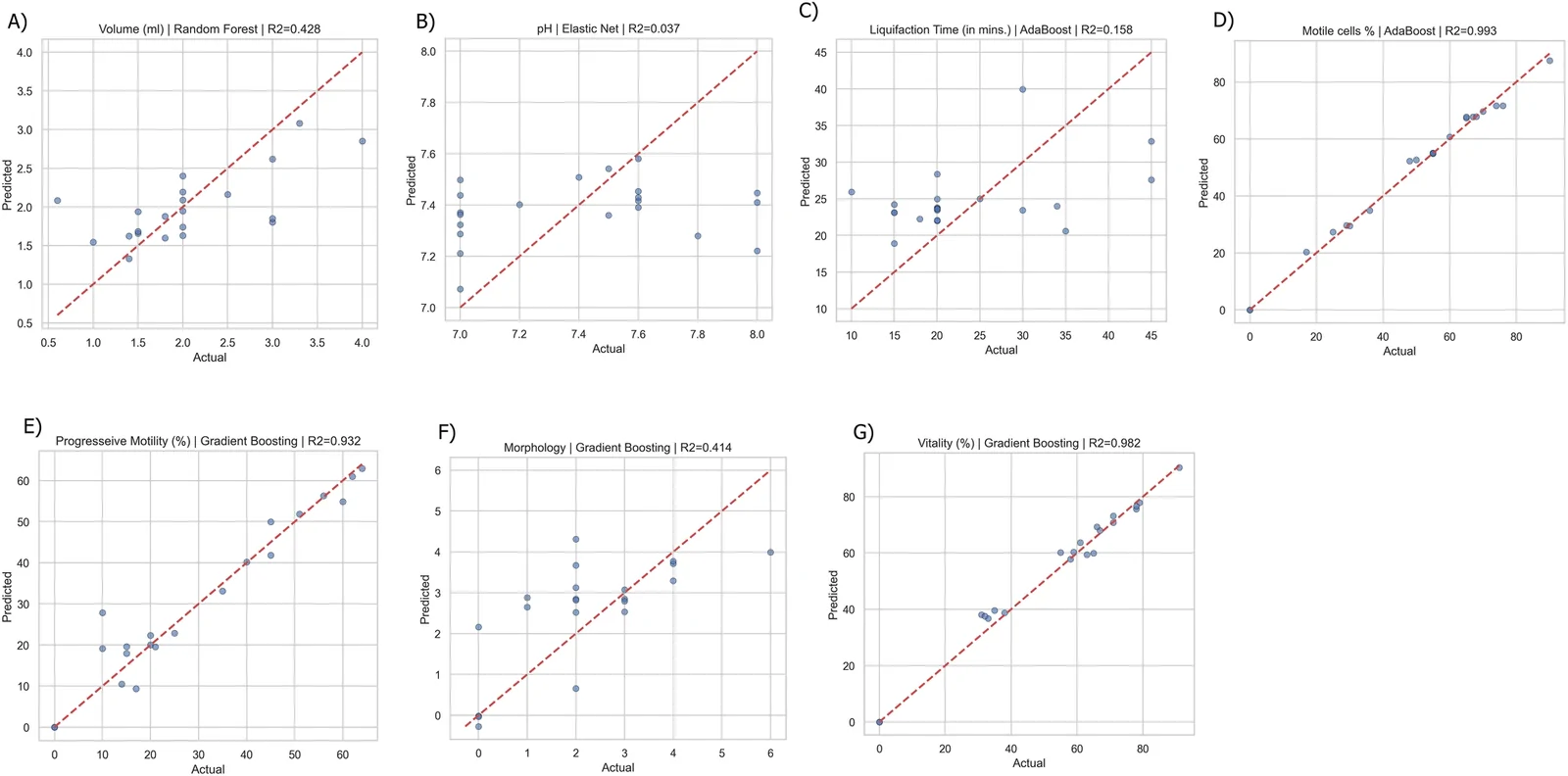
Scatter plots compare actual and predicted observations for semen parameters using the best-performing models. The predictive performance of the best model for each parameter is shown against the ideal fit (dashed red line) and measured with the coefficient of determination (R^2^). **(A)** The Random Forest model predicted semen volume with moderate performance (R^2^ = 0.428); **(B)** The Elastic Net model predicted seminal pH with very low performance (R^2^ = 0.037), with predicted values clustered within a narrow range; **(C)** The AdaBoost model predicted liquefaction time with low performance (R^2^ = 0.158), showing limited agreement between predicted and observed values and wide scatter; **(D)** The AdaBoost model predicted total sperm motility with high performance (R^2^ = 0.993), and predicted values closely matched to the observed values; **(E)** The Gradient Boosting model predicted progressive motility with strong performance (R^2^ = 0.932), and values were closely distributed along the ideal fit; **(F)** The Gradient Boosting model predicted sperm morphology with moderate performance (R^2^ = 0.414), showing noticeable scatter around the regression line; **(G)** The Gradient Boosting model achieved strong performance (R^2^ = 0.982) and predicted values closely matched to the observed measurements.

For semen volume, the Random Forest model showed moderate performance, with an R^2^ of 0.428. Predicted values followed the general trend of observed values, with some scatter around the regression line. For semen pH, the Elastic Net model had very low predictive performance, with an R^2^ of 0.037. Predicted values were clustered within a narrow range, even though observed values varied. For liquefaction time, the AdaBoost model performed poorly, with an R^2^ of 0.158. There was limited agreement between predicted and observed values, along with wide scatter. However, for total sperm motility, the AdaBoost model excelled, with an R^2^ of 0.993. Predicted values closely matched observed values, with most overlapping and aligning with the regression line. For progressive motility, Gradient Boosting achieved strong predictive accuracy (R^2^ = 0.932). For sperm morphology, the Gradient Boosting model showed moderate performance, with an R^2^ of 0.414. It captured the general trend of the data but had noticeable scatter around the regression line. The Gradient Boosting model excelled in predicting sperm vitality, achieving an R^2^ of 0.982. The predicted values closely matched the actual values (Figure 8). Scatter plots compared the observed and predicted values for the best-performing models for each hormonal parameter. For FSH, the AdaBoost model had low predictive performance, with an R^2^ of 0.178. The observations were widely scattered and mostly deviated from the prediction line. For LH, the Random Forest model showed moderate predictive accuracy (R^2^ = 0.432). The predicted values followed the trend of the actual observations, but there was significant dispersion around the regression line. For PRL, the Random Forest model reported an R^2^ of 0.586; however, the scatter plot showed poor alignment. Despite the wide variation in actual PRL levels, the predicted values clustered horizontally within a narrow range, far below the ideal fit.

**Figure 9:**
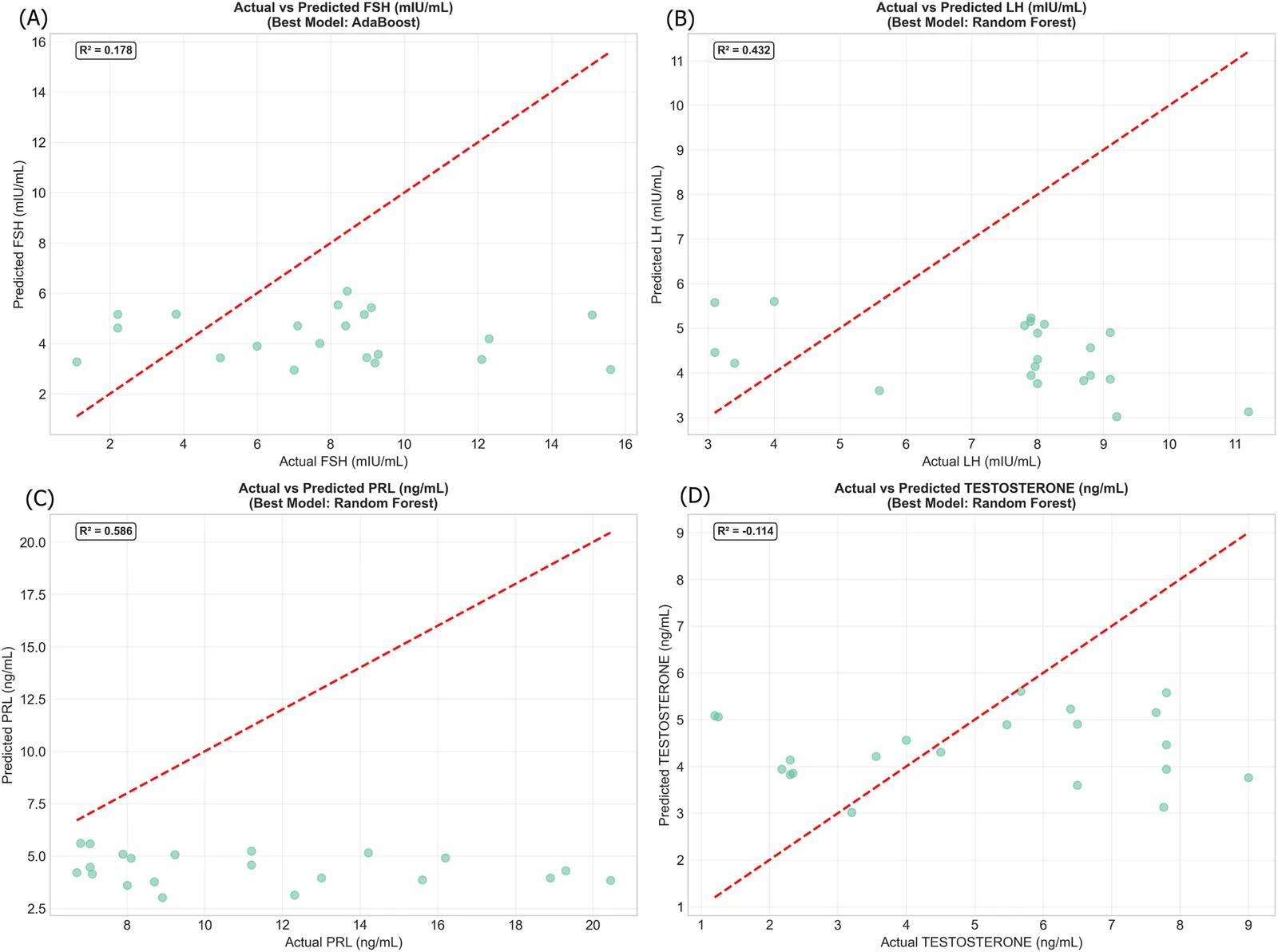
Scatter plots show actual measured concentrations compared to predicted values for reproductive hormones using the best models. **(A)** Follicle-stimulating hormone (FSH) prediction with AdaBoost had low predictive accuracy. The observations were widely scattered around the prediction line, resulting in a low coefficient of determination (R^2^ = 0.178); **(B)** LH prediction with Random Forests demonstrated moderate predictive performance (R^2^ = 0.432); **(C)** PRL prediction with Random Forest showed the highest predictive performance among the studied hormones (R^2^ = 0.586). However, predicted values were concentrated in a narrow range compared to the broader distribution of observed concentrations; **(D)** Testosterone prediction with the Random Forest model performed poorly (R^2^ = −0.114), with clear deviation between observed and predicted values. The red line indicates the line of perfect agreement between observed and predicted concentrations.

This variability highlights the biological heterogeneity inherent in the dataset and indicates that R^2^ captures the total variance explained rather than individual prediction errors. Therefore, the small scatter in the plots does not undermine the model's strong overall predictive performance. The Random Forest model performed poorly in predicting testosterone, with an R^2^ of −0.114 (Figure 9).

### Feature importance

**Figure 10:**
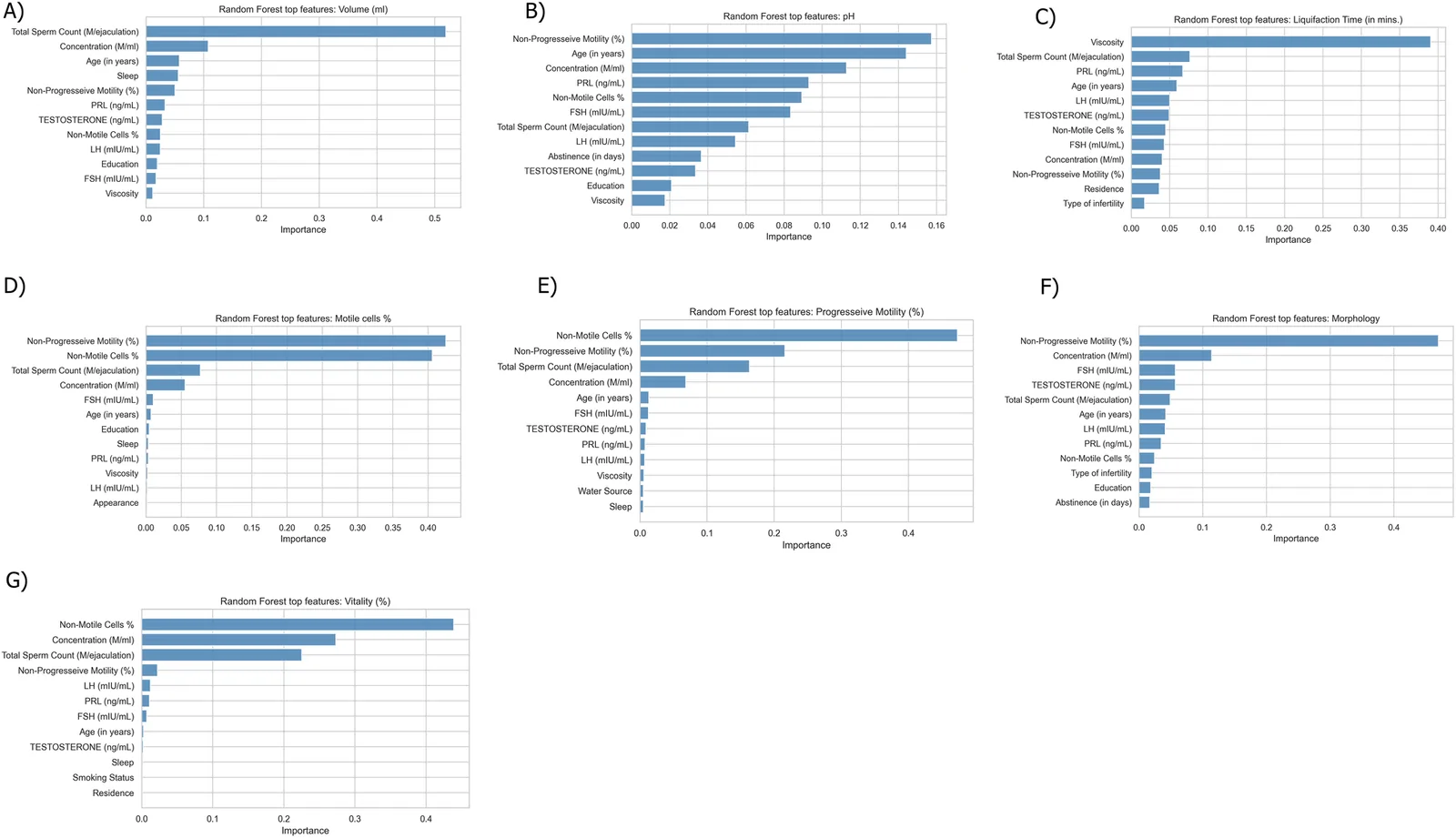
Random forest feature importance scores identify the most influential clinical variables for predicting individual semen parameters. The relative contributions of various clinical and demographic features to the model's predictive accuracy were assessed. **(A)** Semen volume prediction was most strongly influenced by total sperm count (∼0.48), followed by sperm concentration, while age, sleep duration, and hormonal factors contributed modestly; **(B)** Seminal pH prediction was influenced by non-progressive motility, age, sperm concentration, and PRL; **(C)** Liquefaction time prediction identified semen viscosity as the most important feature (≈0.39); **(D)** Sperm motility prediction showed non-progressive motility as the strongest predictor, with total sperm count and sperm concentration contributing to a lesser extent; **(E)** Progressive sperm motility prediction was dominated by non-motile spermatozoa, while hormonal factors contributed negligibly; **(F)** Sperm morphology prediction was strongly influenced by non-progressive motility (∼0.46), followed by concentration and follicle-stimulating hormone (FSH), with other factors showing minor effects; **(G)** Sperm vitality prediction exhibited non-motile cells as the most influential feature (∼0.44), followed by concentration and total sperm count, while hormonal and demographic variables showed very limited importance.

**Figure 11:**
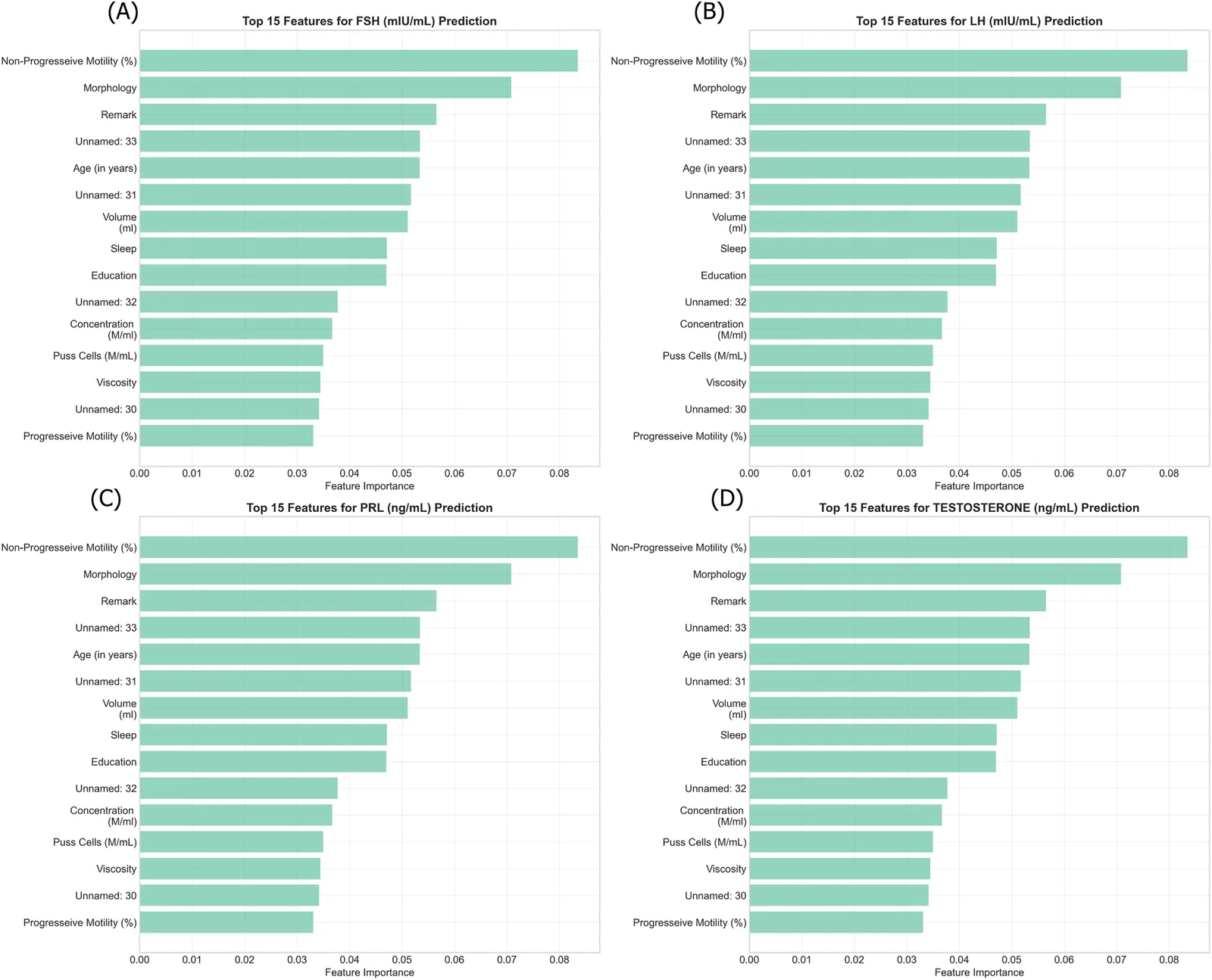
Feature importance analysis of predictors related to hormonal parameter prediction. Bar plots show the relative importance of variables for predicting: **(A)** follicle-stimulating hormone (FSH), **(B)** luteinizing hormone (LH), **(C)** prolactin (PRL), and **(D)** testosterone levels. **(A)** In predicting FSH, non-progressive motility was the most important, followed by morphology and age. Semen volume, sleep duration, and educational level were moderately important, while non-motile cells, alcohol consumption, and water source were the least important; **(B)** A similar trend emerged for LH prediction. Here too, non-progressive motility was the strongest predictor, followed by morphology and age. Semen volume, sleep duration, and educational level had moderate importance, while alcohol consumption and drinking water source had minor influence; **(C)** For PRL prediction, non-progressive motility remained the most significant feature, followed by morphology and age. Concentration, pus cells, viscosity, progressive motility, total sperm count, and liquefaction time played moderate roles, whereas non-motile cells, alcohol consumption, and water source were the least important; **(D)** In testosterone prediction, a similar ranking appeared with non-progressive motility, morphology, and age as top predictors. Semen volume, sleep duration, and educational level were moderately important, while non-motile cells, alcohol consumption, and water source were least important. Higher feature importance values indicate a greater contribution of each variable to model prediction.

Total sperm count was the most important predictor of semen volume, with an importance score of approximately 0.48. Sperm concentration ranked second in importance, while age, sleep duration, non-progressive motility, PRL, and testosterone contributed to a lesser extent. Seminal pH was influenced by non-progressive motility, age, concentration, and PRL levels. For liquefaction time, semen viscosity was the most important feature (∼0.39), whereas PRL, age, LH, testosterone, and FSH contributed less. The strongest predictor of sperm motility was non-progressive motility, while total sperm count and concentration contributed moderately. Non-motile spermatozoa were the dominant feature for predicting progressive motility, whereas FSH, testosterone, PRL, and LH contributed negligibly. On the other hand, sperm morphology prediction was strongly influenced by non-progressive motility, with the highest importance score (∼0.46), followed by concentration and FSH, while testosterone, sperm count, age, LH, PRL, and non-motile cells showed relatively minor effects. For vitality prediction, non-motile cells were the most influential feature (∼0.44), followed by concentration and total sperm count. Non-progressive motility contributed modestly, whereas hormonal factors such as LH, PRL, FSH, testosterone, and demographic variables showed very limited importance (Figure 10). Feature importance analysis revealed highly similar rankings of predictors across the FSH, LH, PRL, and testosterone prediction models (Figure 11). Non-progressive motility consistently ranked as the most significant predictor across all hormonal parameters, with feature importance scores ranging from 0.083 to 0.084. Sperm morphology was the second most important predictor across all models, with importance values of approximately 0.071, while age ranked third, with scores ranging from 0.053 to 0.054. For FSH prediction, non-progressive motility had the highest predictive importance (0.084), followed by morphology and age (0.071 and 0.054, respectively). Semen volume contributed moderately to model performance, with an importance score of 0.051, while sleep duration and educational level were comparable, each with an importance score of 0.047. Concentration (∼0.037), pus cells (∼0.035), viscosity (∼0.034), progressive motility (∼0.033), total sperm count (∼0.033), and liquefaction time (∼0.032) showed moderate predictive influence. Non-motile cells (∼0.024), alcohol consumption (∼0.022), and water source (∼0.020) were the least important predictors. A similar feature was observed for LH prediction. Non-progressive motility remained the strongest predictor (∼0.083), followed by morphology (∼0.071) and age (∼0.053). Semen volume (∼0.051), sleep duration (∼0.047), and educational level (∼0.047) also demonstrated moderate importance. Similarly, sperm concentration (∼0.037), presence of pus cells (∼0.036), viscosity (∼0.035), progressive motility (∼0.033), total sperm count (∼0.032), and liquefaction time (∼0.032) contributed moderately to prediction performance. The lowest importance values were observed for non-motile cells (∼0.024), alcohol consumption (∼0.022), and water source (∼0.021). In the PRL prediction, non-progressive motility had the highest importance score (∼0.084), followed by sperm morphology (∼0.071) and age (∼0.054). Semen volume (∼0.051), sleep duration (∼0.047), and educational level (∼0.047) remained among the better secondary predictors. Sperm concentration (∼0.037), pus cells (∼0.035), viscosity (∼0.034), progressive motility (∼0.033), total sperm count (∼0.033), and liquefaction time (∼0.032) displayed moderate contributions. Non-motile cells (∼0.024), alcohol consumption (∼0.022), and drinking water source (∼0.020) contributed the least to model prediction. Testosterone prediction exhibited an identical pattern. Non-progressive motility was the strongest predictor (∼0.083), followed by sperm morphology (∼0.071) and age (∼0.053). Semen volume (∼0.051), sleep duration (∼0.047), and educational level (∼0.047) showed notable importance, while concentration (∼0.037), pus cells (∼0.035), viscosity (∼0.034), progressive motility (∼0.033), total sperm count (∼0.032), and liquefaction time (∼0.032) showed moderate contributions. Non-motile cells (∼0.024), alcohol consumption (∼0.022), and drinking water source (∼0.021) had the lowest importance scores (Figure 11).

## Discussion

The assessment of male fertility potential has traditionally relied on semen analysis, which remains the principal method for evaluating a man's reproductive function. Semen analysis encompasses both macroscopic and microscopic seminal parameters, including semen volume, sperm concentration, total motility, progressive motility, morphology, and vitality. These parameters are routinely measured to identify potential causes of male infertility (16). Beyond conventional semen analysis, the assessment of a panel of reproductive hormones such as FSH, LH, PRL, and testosterone is critical, particularly when investigating cases of idiopathic or unexplained infertility (17). Recent advancements in biomedical research, facilitated by Industrial Revolution 4.0, have incorporated artificial intelligence (AI) and, more specifically, ML for predictive analytics. These AI-driven approaches have enabled the elucidation of complex and nonlinear interactions among clinical variables—relationships that often elude traditional statistical analyses (13). As a result, ML-based predictive models have emerged as promising tools for enhancing diagnostic accuracy and supporting clinical decision-making in reproductive medicine. The present study has utilized diverse ML algorithms to assess male infertility and construct a robust predictive model aimed at optimizing diagnostic precision. The mean semen volume (2.06 ± 0.98 mL) exceeded the World Health Organization (WHO) reference value of 1.4 mL, indicating that the study population generally lacked evidence of accessory gland dysfunction or ejaculatory abnormalities. The mean seminal pH observed was consistent with the physiological range recommended by the WHO (16). The mean liquefaction time also fell within WHO-accepted parameters, reflecting normal breakdown of the seminal coagulum and appropriate accessory gland function. Sperm concentration varied widely among participants (40.55 ± 38.72 million/mL), highlighting heterogeneity in spermatogenic efficiency. While the mean sperm concentration surpassed the lower WHO reference threshold, the broad dispersion suggests that a subset of individuals may have compromised spermatogenesis (16). Total sperm motility and progressive motility are pivotal for effective fertilization, as they facilitate the successful passage of sperm through the female reproductive tract. In this cohort, total sperm motility and progressive motility were relatively low and showed inter-individual variability. Reduced progressive motility may be attributable to factors such as oxidative stress, compromized sperm membrane integrity, or altered seminal plasma composition. These findings indicate that impaired sperm motility could constitute a primary contributor to reduced male fertility potential. Moreover, sperm morphology and vitality were suboptimal, with a mean normal morphology of 2.65 ± 1.82%, which is below the WHO reference cut-off for normal spermatozoa (16). Aberrant sperm morphology is closely linked to impaired fertilization, suboptimal sperm-oocyte interaction, and diminished embryonic development (18). Similarly, sperm vitality exhibited moderate reduction and pronounced variability, suggesting a substantial proportion of spermatozoa with compromized membrane integrity (1, 16). Collectively, these results underscore that conventional semen parameters may be insufficient to fully elucidate reproductive dysfunction, especially in cases of idiopathic or unexplained infertility. The mean serum FSH concentration was 7.88 ± 4.03 mIU/mL, indicative of compensatory pituitary activity in response to impaired spermatogenesis. Elevated FSH is commonly associated with diminished germ cell activity and seminiferous tubule dysfunction (19, 20). The mean LH was 7.39 ± 2.59 mIU/mL, indicating preserved hypothalamus-gonadal axis functioning. In contrast, the mean PRL level of 10.64 ± 4.79 ng/mL was consistent with normal gonadal function (21). The mean testosterone concentration (4.47 ± 2.27 ng/mL) fell within the normative range; however, the considerable inter-individual variation implies that some participants may have borderline androgen deficiency. Testosterone is essential for spermatogenesis, the development of secondary sexual characteristics, and the maintenance of overall reproductive health.

In this study, ML-based algorithms, particularly Random Forest, Gradient Boosting, and AdaBoost, significantly outperformed traditional linear and kernel-based methods in predicting semen quality parameters and reproductive hormone levels. The results underscore that male reproductive physiology is governed by complex, nonlinear relationships that are more effectively modelled by ensemble-based predictive frameworks than by conventional statistical regression approaches. Notably, exceptionally high predictive accuracy was achieved for sperm motility (R^2^ = 0.993), vitality (R^2^ = 0.982), and progressive motility (R^2^ = 0.932), likely due to strong biological links among these semen parameters. These semen parameters are characterized by nonlinear interaction patterns (22). A high correlation existed between progressive motility and total sperm motility (r = 0.87), along with significant connections between sperm concentration, total sperm count, and motility, suggesting shared physiological factors. However, the study's modest sample size warrants caution in interpreting these results. Despite using cross-validation, hyperparameter tuning, and independent test-set validation to minimize overfitting, some optimistic bias in performance cannot be entirely ruled out. Therefore, validation on large, multicenter datasets is essential before incorporation into the regular workup algorithm of clinicians. However, for semen volume and sperm morphology, only moderate predictive accuracy was attained: Random Forest achieved moderate capability for semen volume (R^2^ = 0.428), and Gradient Boosting showed similar performance for sperm morphology (R^2^ = 0.414). These outcomes suggest that male infertility is a multifactorial condition and that the presence of latent, unmeasured determinants in the dataset may have limited the predictive capacity of the ML algorithms. In contrast, for seminal pH and liquefaction time, all algorithms exhibited poor predictive performance, with the best models yielding low coefficients of determination for pH (R^2^ = 0.037) and liquefaction time (R^2^ = 0.158). This indicates that these seminal properties are governed by highly variable biochemical processes that are only weakly associated with the clinical variables measured in the study. Models predicting hormone levels performed substantially worse than those predicting semen quality. Among the hormonal parameters, PRL demonstrated the strongest predictive power, with Random Forest achieving an R^2^ of 0.586. LH showed moderate predictive accuracy (R^2^ = 0.432), whereas FSH and testosterone showed poor predictive capability. Testosterone prediction, in particular, was unsuccessful across all models, which produced negative R^2^ values. This underscores the complex and multifactorial regulation of endocrine function in male reproductive health, and shows that the model's effectiveness was parameter-dependent and therefore, optimization and external validation are warranted. A recent study reported that an Elastic Net-based model achieves superior predictive performance for evaluating male fecundity, indicating the effectiveness of ML algorithms (13). Similarly, Ory and colleagues demonstrated that Random Forest is effective at predicting improvement in sperm concentration following varicocele repair. The model achieved good predictive performance (AUC = 0.72) by integrating clinical, seminal, and hormonal data, highlighting the ability of ML algorithms to capture complex, non-linear relationships that traditional statistical methods may miss (12). Moreover, the predictive models do not take into account the factors such as genetic variations, metabolomics, oxidative stress markers and environmental exposures Therefore, further multicentric studies including multidimensional variables and larger sample size are required to understand the model's performance more effectively. As the dataset contains no missing values, potential biases related to missing data were avoided, ensuring that all observations were available for both model development and evaluation. Feature selection helped reduce redundancy, remove irrelevant predictors, and improve interpretability. By focusing on the most relevant variables, the models became less affected by multicollinearity and noise, enhancing computational efficiency and predictive accuracy. To reduce the risk of overfitting, the dataset was split into training and testing sets in an 80:20 ratio, with model development occurring only on the training data and the final evaluation on the independent test set. Hyperparameters were tuned through cross-validation within the training set, and algorithm-specific regularization techniques were applied when appropriate. Despite these measures, there remains a possibility of optimistic performance estimates, and hence, external validation with large, multicenter data is necessary to confirm the models' robustness and generalizability.

Male infertility is a complex disorder influenced by environmental, metabolic and lifestyle factors, where conventional statistical analysis often fails to interpret the nonlinear and multidimensional interactions among the variables (23, 24). However, applying ML algorithms to predict semen quality and hormones, the present study highlights that AI-based models can successfully identify hidden patterns and improve predictive assessment in male infertility management. From this study, Random Forest, Gradient Boosting, and AdaBoost emerged as highly effective predictive tools for semen parameters, including total sperm motility, progressive motility, and vitality, underscoring the potential to develop advanced computational systems to support fertility assessment using routinely available clinical and laboratory data. Implementation of such approaches may reduce diagnostic uncertainty, enhance clinical decision-making, and enable earlier identification of individuals at risk for impaired fertility. Moreover, the poor predictive performance for reproductive hormones indicates that the reproductive endocrine system is a complex biological process that cannot be interpreted with the present variables alone, reinforcing the need to incorporate advanced biomarkers, such as proteomics, metabolomics, oxidative stress markers, and genetic and epigenetic variables. From a clinical perspective, this study supports the growing role of AI- and ML-based algorithms in reproductive medicine, which may aid clinicians improve infertility screening, treatment planning, and the cost-effective management of male infertility by incorporating them into their regular workup algorithm. The main limitation of this study is its relatively modest sample size, which may affect the statistical power, stability, and generalizability of the ML algorithms. Despite the relatively small sample size, the study remains valuable as it compares multiple ML algorithms and employs rigorous validation procedures, ensuring a fair evaluation of the models. This in-principle study provides important insights into predictive relationships, model behaviour, and feature importance. The findings can serve as a benchmark for future research in this field. Consequently, additional research with larger, more diverse datasets and external validation using independent data is needed to verify the robustness, reproducibility, and clinical usefulness of the proposed predictive model.

## Conclusions

The present study demonstrates that semen quality and reproductive hormone parameters exhibit considerable variability and intricate interrelationships that can be systematically investigated using ML approaches. Ensemble learning techniques, such as Random Forest, Gradient Boosting, and AdaBoost, showed comparatively better performance in predicting several semen quality attributes within this dataset. However, these findings are preliminary, therefore, further investigation is required with larger, mutlicentric and independent datasets prior to clinical application. Notably, high predictive accuracy was observed for total sperm motility, progressive motility, and vitality, whereas moderate predictive performance was observed for semen volume, morphology, LH, PRL, pH, liquefaction time, and FSH. This parameter-dependentmodel effectiveness highlights the need for further investigation to enhance predictive accuracy and generalizability. Analyses of feature importance indicated that total sperm count, sperm concentration, and the proportions of non-progressive and non-motile sperm cells were the principal determinants of semen characteristics, whereas reproductive hormones exerted comparatively minimal predictive influence. These findings underscore the potential of ensemble ML frameworks in reproductive medicine to elucidate clinically significant patterns and enhance predictive fertility assessment. Nevertheless, future investigations incorporating genetic, environmental, and lifestyle factors, as well as oxidative stress markers, may further augment predictive robustness and clinical relevance.

## Data Availability

The original contributions presented in the study are included in the article/Supplementary Material, further inquiries can be directed to the corresponding author.
