# Supplementary material for: Comparative machine learning analysis identifies random forest and adaboost as superior models for the evaluation of semen quality and reproductive hormones
Source: Front Reprod Health. 2026 Aug 6;8:1908391. doi: 10.3389/frph.2026.1908391 (PMC13490179; doi:10.3389/frph.2026.1908391)
Supplement: Supplementary file 1 [file Table1.docx]

Supplementary Material

# Supplementary Table S1: Descriptive statistics of semen parameters and male reproductive hormones.

| **Variables** | **Values**  **(mean ± standard deviation)** |
| --- | --- |
| Volume (ml) | 2.06±0.98 |
| Liquefaction Time (in mins.) | 24.97±10.41 |
| pH | 7.41±0.38 |
| Vitality (%) | 54±23.17 |
| Total Sperm Motility (%) | 47.5±23.63 |
| Concentration (M/ml) | 40.55±38.72 |
| Total Sperm Count (M/ejaculate) | 90.75±111.71 |
| Progressive Motility (%) | 29.52±19.43 |
| Morphology (%) | 2.62±1.83 |
| FSH (mIU/mL) | 7.88±4.03 |
| LH (mIU/mL) | 7.39±2.59 |
| PRL (ng/mL) | 10.64±4.79 |
| Testosterone (ng/mL) | 4.47±2.27 |
